# Prevalence of borderline elevated and elevated cholesterol among new adult patients from 23 hospitals in 12 cities of Jiangsu Province: a multicenter cross-sectional study

**DOI:** 10.3389/fnut.2026.1800853

**Published:** 2026-04-01

**Authors:** Wenjing Zhang, Kangting Tang, Li Zhou, Xianghua Ma, Yan Zhao

**Affiliations:** 1 Department of Clinical Nutrition, The Fifth Clinical Medical College of Henan University of Chinese Medicine (Zhengzhou People's Hospital), Zhengzhou, Henan, China; 2 Department of Cardiology, Jiangsu Province Hospital, The First Affiliated Hospital of Nanjing Medical University, Nanjing, Jiangsu, China; 3 Department of Clinical Nutrition, The First Affiliated Hospital of Soochow University (First People's Hospital of Suzhou), Suzhou, Jiangsu, China; 4 Department of Clinical Nutrition, Jiangsu Province Hospital, The First Affiliated Hospital of Nanjing Medical University, Nanjing, Jiangsu, China

**Keywords:** borderline elevated cholesterol, cholesterol, elevated cholesterol, multicenter study, prevalence

## Abstract

**Objective:**

To investigate the prevalence of borderline elevated total cholesterol (TC) and elevated cholesterol, and to examine TC levels across different population groups among new adult inpatients in Jiangsu Province.

**Methods:**

A multi-center cluster sampling method was employed to randomly select 23 hospitals from 12 cities in Jiangsu Province between October 2020 and June 2021, with 200 patients randomly selected from each hospital. Serum TC concentrations were determined using the homogeneous method.

**Results:**

A total of 4503 patients participated in the survey. The overall prevalence of borderline elevated TC and elevated cholesterol was 24.9%. The prevalence was 22.1% in males, significantly lower than the 28.7% observed in females. Age-specific prevalence followed an inverted U-shape, ranging from 11.2% (80–99 years) to a peak of 31.6% (45–49 years), with intermediate rates such as 15.7% (18–24) and 29.9% (60–64). Females and residents of Southern, Central, and Northern Jiangsu had respective risks 1.37, 1.57, 1.45, and 1.54 times higher for developing borderline elevated TC and elevated cholesterol. Patients with neurological and circulatory diseases had a 42% and 19% lower risk of developing borderline elevated TC and elevated cholesterol, respectively, with all *P*-values < 0.05.

**Conclusion:**

The study found that among newly hospitalized adult patients in Jiangsu Province, the prevalence of borderline-high and high TC followed an inverted U-shaped pattern with age—rising at first and then falling—and was lower than the national levels reported. The prevalence of borderline-high and high TC was significantly associated with sex, region, and comorbid neurological and circulatory system diseases.

## 1 Introduction

Metabolic diseases pose a growing and substantial challenge to health systems worldwide (1, 2), with dyslipidemia among the most prevalent conditions. Total cholesterol (TC), a principal lipid marker, independently predicts ASCVD risk. The Global Burden of Disease (GBD) 2021 data (released 16 May 2024) estimated DALYs attributable to high TC at 88 million; although the rate of increase has slowed, high TC nevertheless accounted for 3.65 million deaths, ranking third after hypertension and obesity (3). A recent analysis of 200 countries found that in 1980 mean TC and non–HDL-C levels in Chinese adults were relatively low globally and markedly below Western averages; by 2018 these means had reached or exceeded those of some Western countries (4). A 2018 national survey reported the largest increase in the prevalence of hypercholesterolemia (TC ≥6.2 mmol/L) (5). According to recent literature, the prevalence of hyperlipidemia among adults aged 18 and above in China is 42.1%, while the prevalence of hypercholesterolemia (TC) is 8.3% (6). Borderline elevations of TC are insidious, gradual and systemic, often asymptomatic in the early stages; without timely intervention they may progress to overt hypercholesterolemia and adversely affect health. Prior studies have largely examined community-dwelling populations (6, 7), leaving inpatient data scarce and up-to-date prevalence estimates for borderline and elevated TC in Jiangsu Province lacking. This study therefore aims to determine the prevalence of borderline-elevated and elevated TC among newly admitted adult inpatients in Jiangsu Province to inform the development of targeted interventions and public health strategies.

## 2 Subjects and methods

### 2.1 Study subjects

**Figure 1:**
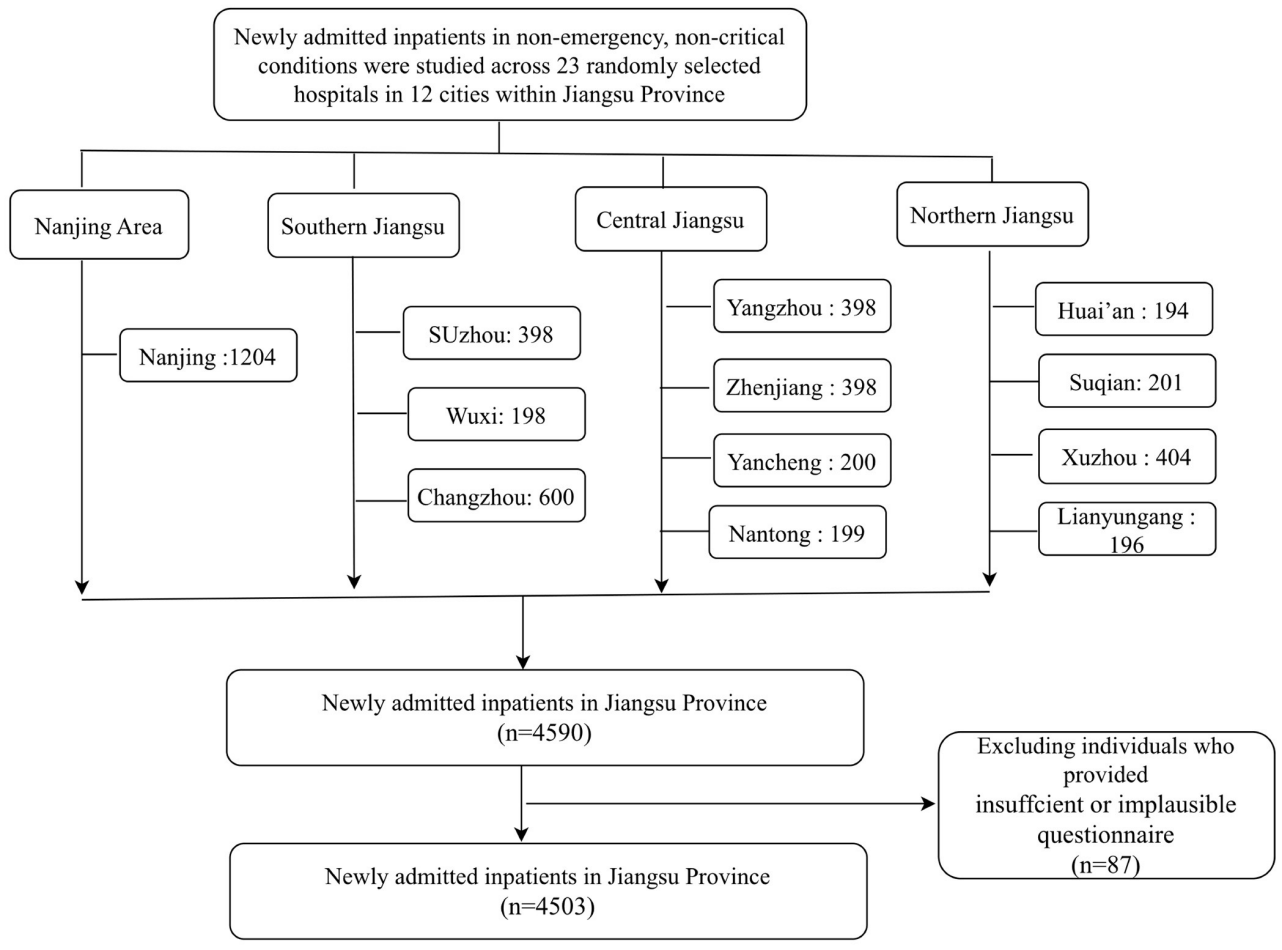
Flowchart of newly admitted inpatients through the study.

The data for this study was sourced from the Jiangsu Province segment of the “China Nutrition Fundamental Data Construction Project” (registration number NCT05694104). The study subjects were newly hospitalized (non-emergency) patients. Inclusion criteria: (i) newly hospitalized patients (non-emergency, non-critical) with one of seven systemic diseases (digestive, respiratory, cardiovascular and endocrine, oncological, neurological, or urological systems); (ii) age ≥18 years; (iii) admission within 24–48 h. Exclusion criteria: (i) pediatric and critical care patients; (ii) those with psychiatric disorders and memory impairment who are unable to answer questions correctly; (iii) individuals lacking capacity for autonomy; (iv) other situations deemed unsuitable for inclusion by the researchers (see Figure 1).

### 2.2 Sampling method

This study is a multicenter cross-sectional investigation, relying on the National Nutrition Basic Database Survey, with Jiangsu Provincial People's Hospital serving as the provincial leading hospital. During the study period (October 2020 to June 2021), 12 cities in Jiangsu Province were selected, with 1 to 2 secondary or higher-level hospitals randomly chosen from each city. A continuous fixed-point sampling method was employed for data collection. The study participants included patients with diseases from seven systems: digestive, respiratory, cardiovascular, endocrine, oncology, neurological, and urinary systems, until the total number of observed cases at each hospital reached 200, with no fewer than 25 cases from each system. The study area was divided into four regions: Nanjing (Nanjing City), Southern Jiangsu (Suzhou, Wuxi, Changzhou), Central Jiangsu (Yangzhou, Zhenjiang, Yancheng, Nantong), and Northern Jiangsu (Huai'an, Suqian, Xuzhou, Lianyungang).This study received ethical approval from the Ethics Committee of Peking Union Medical College Hospital (Approval No.: ZS-2614).

### 2.3 Blood sample collection and testing

Fasting venous blood samples were collected from the patients, with fasting lasting for at least 8 h, from the elbow vein. The blood was centrifuged to obtain serum, which was then stored in a freezer at −80 °C. A professional medical laboratory technician employed the homogeneous assay method using a Beckman Coulter fully automated biochemical analyzer (AU-5800, USA) for batch testing of serum TC concentrations.

### 2.4 Survey methodology

To ensure verification efficiency and traceability, this survey utilized a dual reporting method with both paper and electronic forms. The survey content included demographic information, disease details, physical examinations, body composition measurements, nutritional risk screening, and nutritional assessments. Initially, paper survey forms were completed. Within one week of concluding each case investigation, the data from the paper forms were entered into the China Nutrition Fundamental Database platform.

### 2.5 Quality control

First, a quality control leadership team was established in Jiangsu Province to oversee the entire quality control process of the survey, including sampling, questionnaire surveys, physical examinations, laboratory tests, and data management, in accordance with project-specific quality control standards and methods. Each city, district/county, and hospital survey site appointed designated personnel to ensure quality control throughout various stages of the investigation. Second, prior to the survey, the survey plan was repeatedly reviewed, a unified work plan was developed, and all survey forms and manuals were standardized. Detailed instructions were provided for the use and measurement methods of each type of measuring instrument. Calibrated measuring tools, such as weighing scales, blood pressure monitors, and body composition analyzers, were uniformly supplied. A standardized blood sample testing protocol was also adopted. Third, a comprehensive training plan was developed, and all quality control personnel and investigators were required to undergo training and pass an assessment before participating in the field survey. During the implementation phase, each survey site verified the collected data. After ensuring its accuracy, the data was entered into the computer system using a unified procedure developed for the project, with automatic range and logical sequence settings to ensure accurate data entry. Fourth, external support (CRC) was introduced to provide external supervision and evaluation of the project.

### 2.6 Definitions of indicators

According to the Diagnostic Criteria from the “2016 Chinese guideline for the management of dyslipidemia in adults (in Chinese)” borderline elevated TC was defined as a range of ≥5.2 mmol/L (~200 mg/dL) and < 6.2 mmol/L (~240 mg/dL), while elevated TC was defined as TC ≥6.2 mmol/L (~240 mg/dL) (8).

### 2.7 Statistical analysis

Given the multicenter stratified cluster sampling design employed in this study, observations were nested within hospitals and regions, potentially violating the independence assumption of standard logistic regression. To account for this hierarchical data structure and adjust for clustering effects, we conducted sensitivity analyses using generalized linear mixed models (GLMMs) with random intercepts for hospital. Model fitting was performed using the lme4 and lmerTest packages in R, with restricted maximum likelihood (REML) estimation for variance components and Wald tests for fixed effects.

Statistical analyses were performed using IBM SPSS Statistics version 25.0 (IBM Corp., Armonk, NY, USA). Forest plots were generated using R software (version 4.4; R Foundation for Statistical Computing, Vienna, Austria) with the forestplot package. Line graphs and histograms were created using GraphPad Prism (version 9.0; GraphPad Software, San Diego, CA, USA). Geographic distribution maps were produced using ArcGIS (version 10.2; Esri, Redlands, CA, USA).

Prior to analysis, data were examined for completeness and plausibility. The proportion of missing data for all variables included in the analysis was < 5%. Given the low proportion and absence of systematic patterns, complete-case analysis was performed without imputation.

For continuous variables, normality was assessed using the Shapiro–Wilk test. Normally distributed variables were expressed as mean ± standard deviation (SD) and compared using independent-samples *t* tests. For skewed distribution of continuous data, median (M) and interquartile range (Q1, Q3) were used for description, with differences between groups assessed using non-parametric tests. Categorical variables were summarized as frequencies and percentages (%). Group differences were assessed using the chi-square (χ^2^) test.

Univariate logistic regression analyses were first performed to evaluate potential associations between each independent variable and borderline-elevated/elevated TC. Variables with *P* < 0.05 in univariate analyses, chi-square tests, or rank-sum tests or with established clinical relevance were entered into the multivariable logistic regression model using the enter method. Unadjusted and adjusted odds ratios (ORs) and 95% confidence intervals (CIs) were calculated to estimate the strength of associations.

All statistical tests were two-sided, and a *P*-value < 0.05 was considered statistically significant.

To improve comparability across regions and hospitals with different age structures, age-standardized prevalence rates were calculated using the direct standardization method. The age distribution of the 2020 Chinese national census population was used as the standard population. Variance was estimated using the binomial approximation, and 95% CIs were derived based on the normal distribution.

## 3 Results

### 3.1 Prevalence of borderline elevated tc and hypercholesterolemia in populations with different characteristics

**Figure 2:**
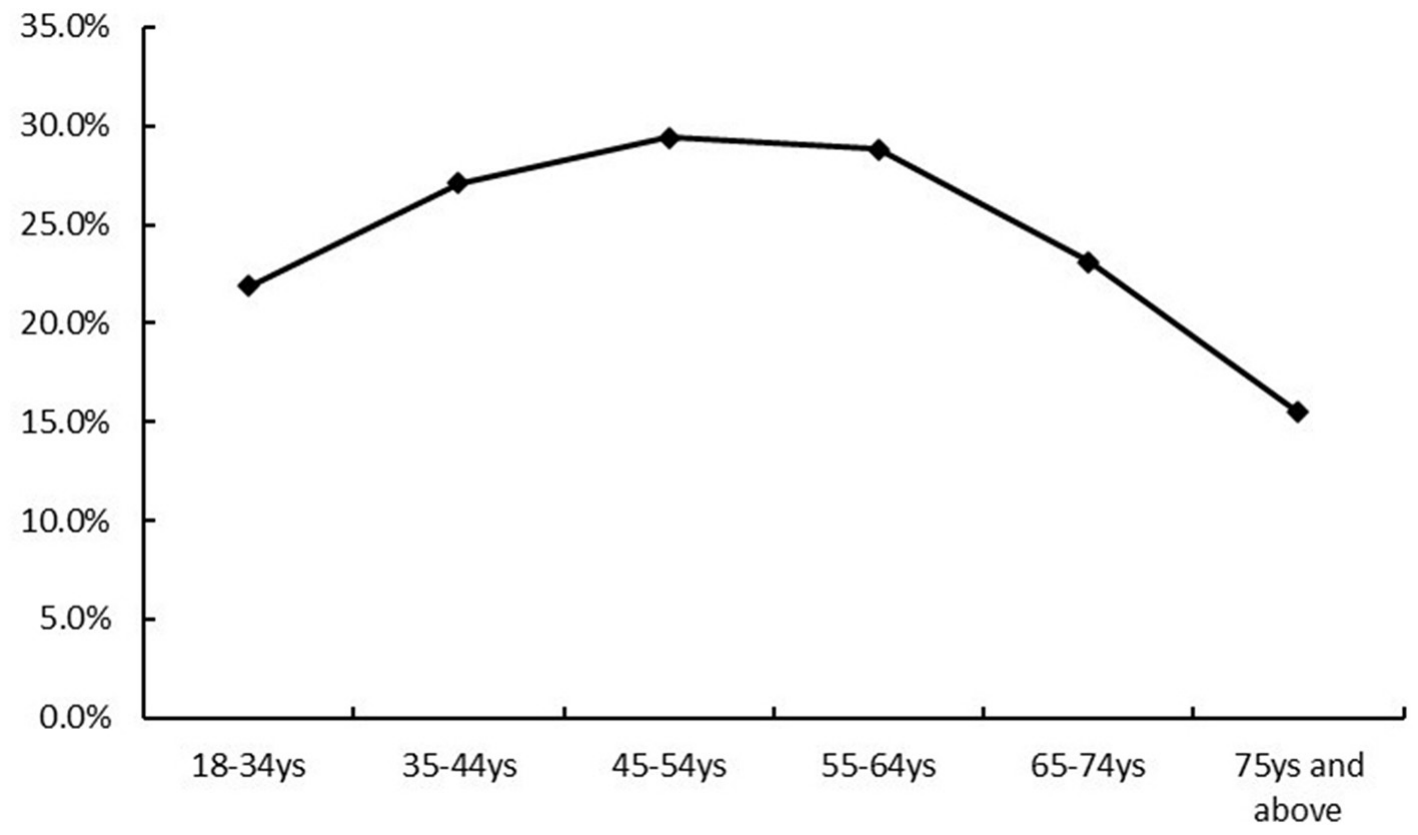
Age Distribution of the prevalence of elevated TC and high TC in Jiangsu Province (line graph).

**Figure 3:**
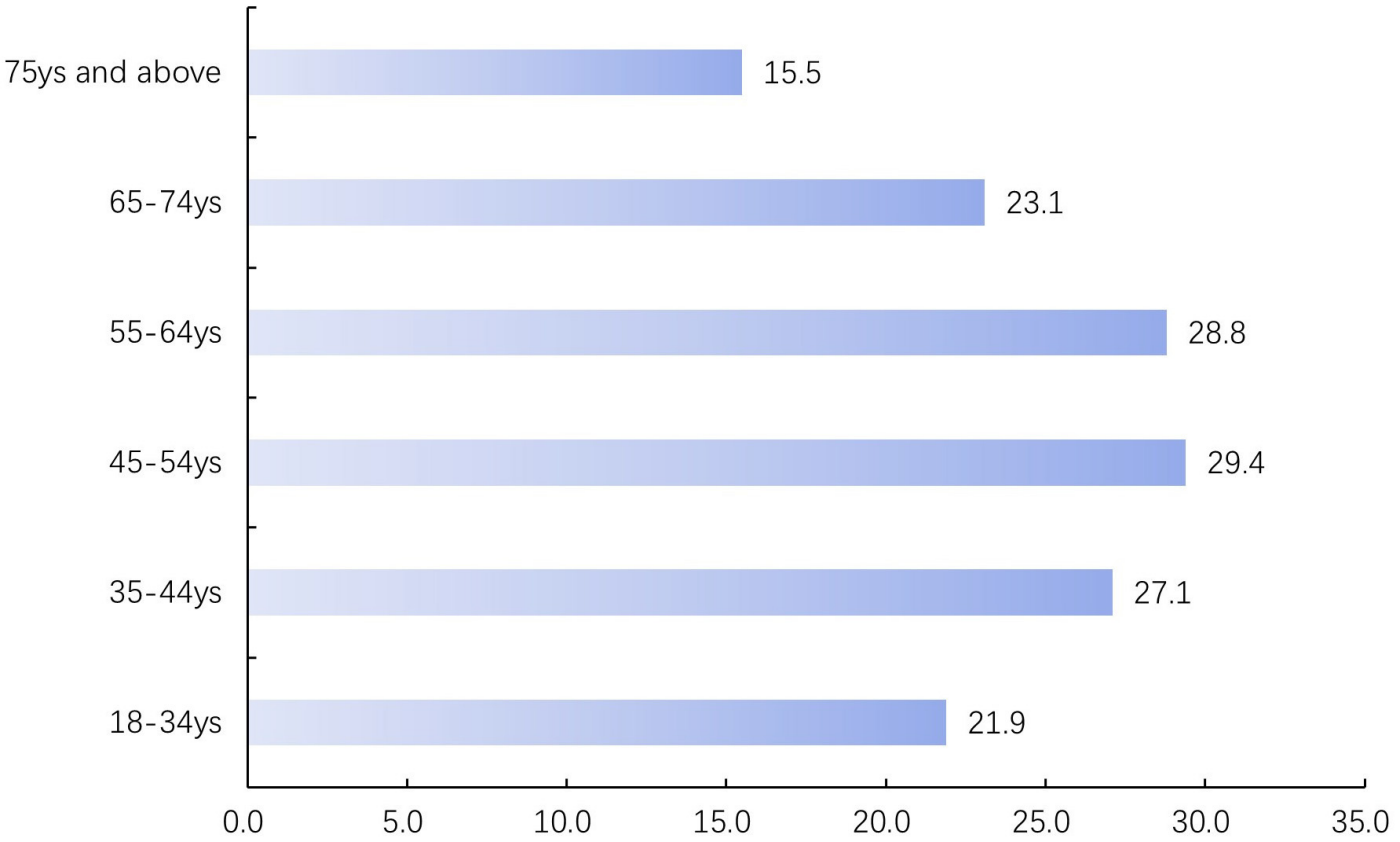
The prevalence of elevated TC levels and hypercholesterolemia.

**Figure 4:**
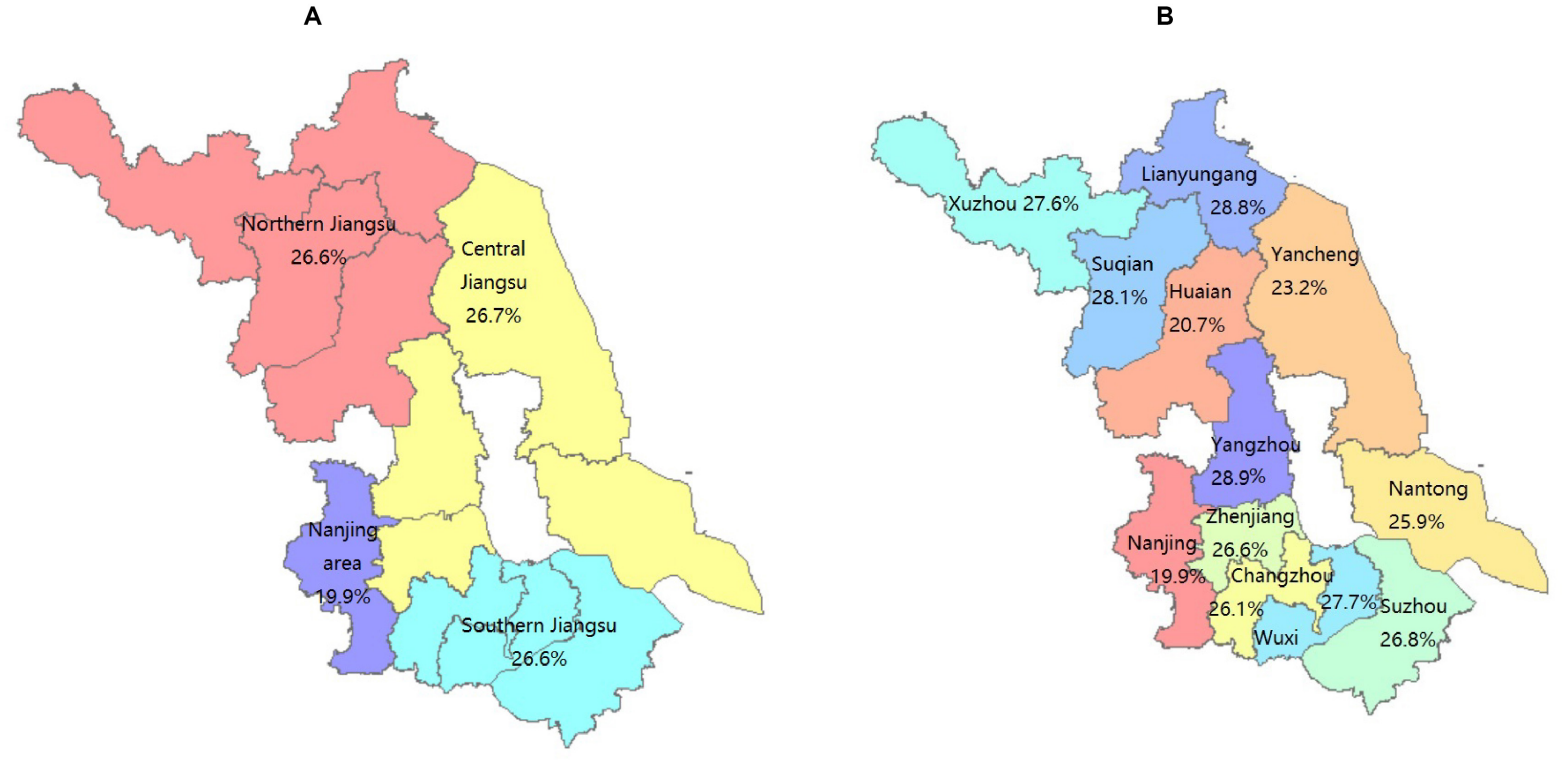
Distribution of the prevalence of elevated TC and high TC in cities and regions of Jiangsu Province. **(A)**. Map of the Regional Distribution of the Prevalence of Elevated TC and High TC in Jiangsu Province **(B)**. Map of the Urban Distribution of the Prevalence of Elevated TC and High TC in Jiangsu Province.

A total of 4,503 adult inpatients were included. The overall prevalence of borderline-elevated or elevated TC was 24.8% (*n* = 1,120), comprising 574 men and 546 women. The prevalence in men was 22.1%, which was significantly lower than in women (28.7%). By diagnostic category, patients with endocrine, nutritional and metabolic diseases (28.1%), neurological diseases (18.1%) and circulatory system diseases (22.1%) had significantly higher prevalences of borderline-elevated/elevated TC than patients without these diagnoses (Table 1). Prevalence by age group was 21.9% (18–34 years), 27.1% (35–44 years), 29.4% (45–54 years), 28.8% (55–64 years), 23.1% (65–74 years) and 15.5% (≥75 years), exhibiting an inverted U-shaped trend with increasing age (Figures 2, 3). The prevalence rates of TC and borderline elevated TC in different regions were as follows: Nanjing region 19.9%, Southern Jiangsu region 26.6%, Central Jiangsu region 26.7%, and Northern Jiangsu region 26.6%. There were significant differences in prevalence rates among these regions (χ^2^ = 21.03, *P* < 0.001). Notably, the prevalence rate in Nanjing was the lowest, while the rates in Southern, Central, and Northern Jiangsu regions were comparable and higher than that of Nanjing, as detailed in Table 1 and Figure 4A. Further analysis of individual cities revealed that the prevalence rates of elevated TC and borderline elevated TC varied as follows: Nanjing 19.9%, Xuzhou 27.6%, Lianyungang 28.8%, Suqian 28.1%, Yangzhou 28.9%, Zhenjiang 26.6%, Yancheng 23.2%, Wuxi 27.7%, Suzhou 26.8%, Changzhou 26.1%, Nantong 25.9%, and Huai'an 20.7%. Significant differences in prevalence rates were also observed among these cities (χ^2^ = 28.16, *P* = 0.003), with Nanjing displaying the lowest prevalence rate in the province, as illustrated in Table 1 and Figure 4B. All observed differences were statistically significant.

**Table 1:** Prevalence of hypercholesterolemia and general TC level characteristics in newly hospitalized adult patients from 23 hospitals across 12 cities in Jiangsu Province.

Characteristics	*N*	TC < 5.2	TC ≥5.2	*χ2*	*p*-Value	TC concentration	*p*-Value
		*N* (%)	*N* (%)			*P*_50_ (*P*_25~_*P*_75_)	
Age, years							
18–34 ys	283	221 (78.1%)	62 (21.9%)	51.84	0.00	4.23 (3.49–4.96)	0.00
35–44 ys	347	253 (72.9%)	94 (27.1%)			4.39 (3.68–5.27)	
45–54 ys	776	548 (70.6%)	228 (29.4%)			4.44 (3.74–5.40)	
55–64 ys	1,203	857 (71.2%)	346 (28.8%)			4.37 (3.63–5.32)	
65–74 ys	1,269	976 (76.9%)	293 (23.1%)			4.21 (3.44–5.03)	
75 ys and above	625	528 (84.5%)	97 (15.5%)			3.98 (3.26–4.77)	
Gender							
Male	2,601	2,027 (77.9%)	574 (22.1%)	25.91	**0.00**	4.18 (3.44–5.03)	**0.00**
Female	1,902	1,356 (71.3%)	546 (28.7%)			4.46 (3.73–5.30)	
Nationality							
Han	4,489	3,373 (75.1%)	1,116 (24.9%)	0.10	0.75	4.30 (3.55–5.16)	0.24
Others	14	10 (71.4%)	4 (28.6%)			4.69 (3.89–5.76)	
Regional distribution							
Nanjing Area	1,180	945 (80.1%)	235 (19.9%)	21.03	**0.00**	4.24 (3.50–4.97)	**0.006**
Southern Jiangsu	1,177	864 (73.4%)	313 (26.6%)			4.37 (3.66–5.23)	
Central Jiangsu	1,168	856 (73.3%)	312 (26.7%)			4.29 (3.55–5.24)	
Northern Jiangsu	978	718 (73.4%)	260 (26.6%)			4.33 (3.50–5.25)	
City distribution							
Nanjing	1,180	945 (80.1%)	235 (19.9%)	28.16	0.003	4.24 (3.50–4.97)	0.006
Xuzhou	395	286 (72.4%)	109 (27.6%)			4.41 (3.58–5.27)	
Lianyungang	191	136 (71.2%)	55 (28.8%)			4.36 (3.30–5.43)	
Suqian	199	143 (71.9%)	56 (28.1%)			4.41 (3.70–5.30)	
Yangzhou	394	280 (71.1%)	114 (28.9%)			4.25 (3.44–5.42)	
Zhenjiang	387	284 (73.4%)	103 (26.6%)			4.40 (3.60–5.23)	
Yancheng	194	149 (76.8%)	45 (23.2%)			4.28 (3.67–5.13)	
Wuxi	195	141 (72.3%)	54 (27.7%)			4.45 (3.60–5.38)	
Suzhou	392	287 (73.2%)	105 (26.8%)			4.25 (3.45–5.23)	
Changzhou	590	436 (73.9%)	154 (26.1%)			4.41 (3.77–5.22)	
Nantong	193	143 (74.1%)	50 (25.9%)			4.08 (3.33–5.20)	
Huai'an	193	153 (79.3%)	40 (20.7%)			4.07 (3.35–4.81)	
Education level							
No schooling	598	462 (77.3%)	136 (22.7%)	1.72	0.42	4.30 (3.53–5.09)	0.73
Primary to High School (Technical secondary school)	3,324	2,485 (74.8%)	839 (25.2%)			4.31 (3.55–5.18)	
Bachelor	576	431 (74.8%)	145 (25.2%)			4.31 (3.62–5.17)	
Diagnosis of infectious and parasitic diseases							
No	4,454	3,343 (75.1%)	1,111 (24.9%)	1.12	0.29	4.31 (3.56–5.17)	**0.014**
Yes	49	40 (81.6%)	9 (18.4%)			4.00 (3.13–5.03)	
Diagnosis of tumor							
No	3,394	2,571 (75.8%)	823 (24.2%)	2.87	0.09	4.29 (3.55–5.13)	0.24
Yes	1,109	812 (73.2%)	297 (26.8%)			4.33 (3.56–5.25)	
Diagnosis of blood and hematopoietic system diseases							
No	4,381	3,283 (74.9%)	1,098 (25.1%)	3.14	0.076	4.31 (3.56–5.17)	**0.026**
Yes	122	100 (82.0%)	22 (18.0%)			4.05 (3.28–4.93)	
Diagnosis of endocrine, nutritional, and metabolic diseases							
No	3,325	2,536 (76.3%)	789 (23.7%)	8.89	**0.003**	4.29 (3.53–5.11)	**0.009**
Yes	1,178	847 (71.9%)	331 (28.1%)			4.36 (3.59–5.31)
Diagnosis of nervous system diseases							
No	3,834	2,835 (73.9%)	999 (26.1%)	19.36	**0.00**	4.35 (3.59–5.22)	**0.00**
Yes	669	548 (81.9%)	121 (18.1%)			4.11 (3.35–4.86)	
Diagnosis of eye, ear, nose, and throat diseases							
No	4,460	3,350 (75.1%)	1,110 (24.9%)	0.061	0.81	4.31 (3.55–5.17)	0.55
Yes	43	33 (76.7%)	10 (23.3%)			4.18 (3.47–5.12)	
Diagnosis of circulatory system diseases							
No	2,741	2,011 (73.4%)	730 (26.6%)	11.62	**0.001**	4.37 (3.60–5.24)	**0.00**
Yes	1,762	1,372 (77.9%)	390 (22.1%)			4.20 (3.44–5.02)	
Diagnosis of respiratory system diseases							
No	3,761	2,822 (75.0%)	939 (25.0%)	0.11	0.74	4.32 (3.57–5.17)	0.18
Yes	742	561 (75.6%)	181 (24.4%)			4.24 (3.44–5.15)	
Diagnosis of digestive system diseases							
No	3,662	2,756 (75.3%)	906 (24.7%)	0.18	0.67	4.29 (3.56–5.16)	0.85
Yes	841	627 (74.6%)	214 (25.4%)			4.37 (3.51–5.19)	
Diagnosis of skin and subcutaneous tissue diseases							
No	4,482	3,366 (75.1%)	1,116 (24.9%)	0.38	0.54	4.31 (3.55–5.17)	0.41
Yes	21	17 (81.0%)	4 (19.0%)			4.04 (3.13–4.95)	
Diagnosis of musculoskeletal system diseases							
No		3,341 (75.1%)	1,108 (24.9%)	0.21	0.65	4.31 (3.55–5.17)	0.79
Yes	54	42 (77.8%)	12 (22.2%)			4.07 (3.63–4.97)	
Diagnosis of immune system diseases							
No	4,479	3,369 (75.2%)	1,110 (24.8%)	3.64	0.056	4.30 (3.55–5.16)	0.055
Yes	24	14 (58.3%)	10 (41.7%)			4.91 (3.91–5.43)	
Diagnosis of genitourinary system diseases							
No	3,795	2,869 (75.6%)	926 (24.4%)	2.88	0.09	4.28 (3.53–5.14)	**0.006**
Yes	708	514 (72.6%)	194 (27.4%)			4.38 (3.72–5.26)	
Diagnosis of malnutrition–related diseases							
No	4,485	3,368 (75.1%)	1,117 (24.9%)	0.65	0.42	4.31 (3.55–5.17)	0.21
Yes	18	15 (83.3%)	3 (16.7%)			3.92 (2.82–4.93)	
Number of diagnosed diseases							
Single Disease	2,349	1,747 (74.4%)	602 (25.6%)	1.5	0.22	4.36 (3.62–5.20)	0.003
Multiple Diseases	2,154	1,636 (76.0%)	518 (24.0%)			4.25 (3.44–5.13)	

*a*. Median test; *b*. Mann-Whitney test. 5.2 mmol/L [~200 mg/dL], 6.2mmol/L [~240mg/dL].

The crude prevalence of borderline elevated and elevated TC varied across regions, ranging from 19.9% in the Nanjing area to 26.7% in Central Jiangsu. After direct age standardization using the 2020 Chinese census population, regional differences persisted. The age-standardized prevalence was: 19.1% (95% CI: 17.0%−21.2%) in the Nanjing area, 25.8% (95% CI: 23.2%−28.4%) in Southern Jiangsu, 27.5% (95% CI: 24.7%−30.3%) in Central Jiangsu, 28.1% (95% CI: 25.1%−31.1%) in Northern Jiangsu. The overall age-standardized prevalence was 24.9% (95% CI: 23.5%−26.3%), which was comparable to the crude prevalence of 25.0%, suggesting minimal distortion due to age structure in the pooled sample. Compared with crude rates, age standardization slightly attenuated the prevalence in Southern Jiangsu but increased estimates in Central and Northern Jiangsu (see Table S1).

### 3.2 TC levels across populations with different characteristics

TC levels by age group were 4.23, 4.39, 4.44, 4.37, 4.21, and 3.98 mmol/L for the 18–34, 35–44, 45–54, 55–64, 65–74, and ≥75 years groups, respectively; these differences were statistically significant (*P* < 0.001). TC was lower in men (4.18 mmol/L) than in women (4.46 mmol/L) (*P* < 0.001). The TC levels across different regions were as follows: Nanjing region 4.24 (3.50–4.97) mmol/L, Southern Jiangsu region 4.37 (3.66–5.23) mmol/L, Central Jiangsu region 4.29 (3.55–5.24) mmol/L, and Northern Jiangsu region 4.33 (3.50–5.25) mmol/L. The differences in TC levels among these regions were statistically significant (*P* = 0.006). Regarding the TC levels in individual cities, the values for Nanjing, Xuzhou, Lianyungang, Suqian, Yangzhou, Zhenjiang, Yancheng, Wuxi, Suzhou, Changzhou, Nantong, and Huai'an were 4.24 (3.50–4.97) mmol/L, 4.41 (3.58–5.27) mmol/L, 4.36 (3.30–5.43) mmol/L, 4.41 (3.70–5.30) mmol/L, 4.25 (3.44–5.42) mmol/L, 4.40 (3.60–5.23) mmol/L, 4.28 (3.67–5.13) mmol/L, 4.45 (3.60–5.38) mmol/L, 4.25 (3.45–5.23) mmol/L, 4.41 (3.77–5.22) mmol/L, 4.08 (3.33–5.20) mmol/L, and 4.07 (3.35–4.81) mmol/L, respectively. The differences in TC levels among these cities were also statistically significant (*P* = 0.006). Patients with infectious diseases, hematological disorders, neurological diseases, circulatory system diseases, or multiple comorbidities had significantly lower TC levels than those without these diagnoses. Conversely, patients with endocrine, nutritional and metabolic disorders or genitourinary diseases had significantly higher TC levels. All intergroup comparisons were statistically significant (all *P* < 0.05) (Table 1).

### 3.3 Multivariate analysis of borderline elevated and elevated tc levels

**Figure 5:**
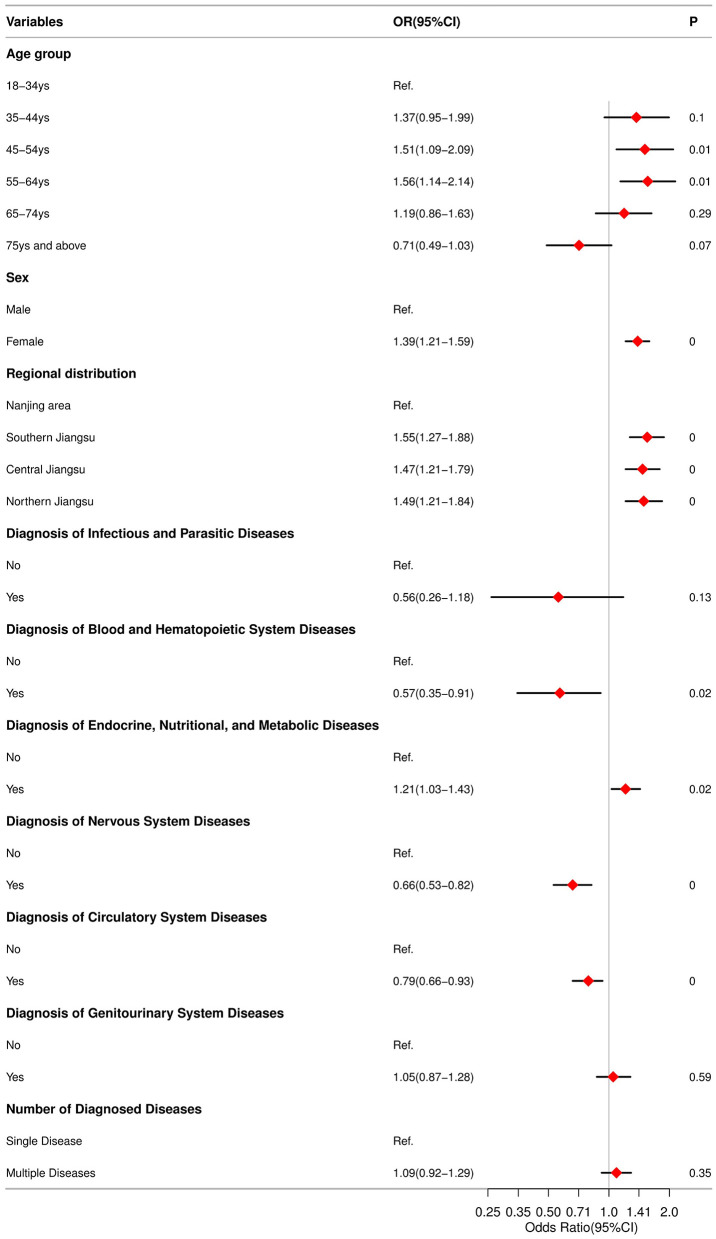
Relationship between variables related to borderline and elevated TC among newly hospitalized adult patients in 23 hospitals across 12 cities in Jiangsu Province.

Univariate analysis showed that age groups 45–54, 55–64, and ≥75 years, female sex, residence in Southern, Central or Northern Jiangsu, and a diagnosis of endocrine, nutritional and metabolic disorders were associated with higher odds of borderline-elevated or elevated TC. In contrast, neurological and circulatory system diseases were associated with lower odds. Using borderline-elevated/elevated TC as the dependent variable (0 = no, 1 = yes), a multivariable logistic regression model including variables significant on univariate analysis was fitted. After adjustment, the following factors remained independently associated with increased odds of borderline-elevated/elevated TC: age 45–54 (OR = 1.51), age 55–64 (OR = 1.56), female sex (OR = 1.39), and residence in Southern Jiangsu (OR = 1.55), Central Jiangsu (OR = 1.47) and Northern Jiangsu (OR = 1.49). Conversely, diagnoses of neurological or circulatory system disease were associated with reduced odds (OR = 0.66 and OR = 0.79, corresponding to reductions of 34% and 21%, respectively). All reported associations were statistically significant (*P* < 0.05). Detailed results were presented in Figure 5.

### 3.4 Results of multilevel modeling analysis

To address potential clustering effects due to the multicenter design, we fitted multilevel logistic regression models with random intercepts for hospital. The estimated variance of the random intercept was 0.0262 (standard error = 0.162), yielding an intraclass correlation coefficient (ICC) of 0.0079. This indicates that approximately 0.79% of the total variance in TC elevation was attributable to between-hospital differences, reflecting weak clustering effects. Fixed-effect estimates from the multilevel model are presented in Table 2. Overall, the direction and magnitude of associations were consistent with those from the standard multivariate logistic regression (see Table 2).

**Table 2:** Logistic mixed-effects regression analysis of borderline elevated and elevated TC levels among newly hospitalized adult patients.

Characteristics	Univariate regression		Multivariate regression	
	OR (*95% CI*)	*p*-Value	OR (*95% CI*)	*p*-Value
Age, years				
18–34 ys	Ref		Ref	
35–44 ys	1.30 (0.90–1.89)	0.158	1.34 (0.92–1.94)	0.124
45–54 ys	1.44 (1.04–1.99)	0.027	1.47 (1.06–2.04)	0.02
55–64 ys	1.42 (1.04–1.93)	0.027	1.49 (1.08–2.04)	0.014
65–74 ys	1.04 (0.76–1.41)	0.828	1.13 (0.82–1.57)	0.441
75 ys and above	0.62 (0.44–0.89)	0.009	0.69 (0.48–0.99)	0.046
Gender				
Male	Ref		Ref	
Female	1.40 (1.22–1.60)	< 0.001	1.40 (1.22–1.61)	< 0.001
Education level				
No schooling	Ref			
Primary to high school (technical secondary school)	1.16 (0.94–1.42)	0.175		
Bachelor	1.21 (0.92–1.59)	0.175		
Diagnosis of infectious and parasitic diseases				
No	Ref			
Yes	0.65 (0.31–1.35)	0.249		
Diagnosis of tumor				
No	Ref		Ref	
Yes	1.20 (1.03–1.41)	0.022	1.17 (0.98–1.39)	0.078
Diagnosis of blood and hematopoietic system diseases				
No	Ref			
Yes	0.63 (0.40–1.01)	0.055		
Diagnosis of endocrine, nutritional, and metabolic diseases				
No	Ref		Ref	
Yes	1.25 (1.08–1.45)	0.004	1.30 (1.11–1.52)	0.001
Diagnosis of nervous system diseases				
No	Ref		Ref	
Yes	0.62 (0.50–0.76)	< 0.001	0.70 (0.57–0.87)	0.002
Diagnosis of eye, ear, nose, and throat diseases				
No	Ref			
Yes	0.93 (0.45–1.89)	0.832		
Diagnosis of circulatory system diseases				
No	Ref		Ref	
Yes	0.78 (0.67–0.89)	< 0.001	0.85 (0.73–0.99)	0.033
Diagnosis of respiratory system diseases				
No	Ref			
Yes	0.98 (0.81–1.17)	0.809		
Diagnosis of digestive system diseases				
No	Ref			
Yes	1.04 (0.88–1.24)	0.65		
Diagnosis of skin and subcutaneous tissue diseases				
No	Ref			
Yes	0.70 (0.24–2.09)	0.527		
Diagnosis of musculoskeletal system diseases				
No	Ref			
Yes	0.90 (0.47–1.72)	0.749		
Diagnosis of immune system diseases				
No	Ref			
Yes	2.22 (0.98–5.03)	0.055		
Diagnosis of genitourinary system diseases				
No	Ref			
Yes	1.16 (0.97–1.39)	0.107		
Diagnosis of malnutrition–related diseases				
No	Ref			
Yes	0.59 (0.17–2.06)	0.412		
Number of diagnosed diseases				
Single disease	Ref			
Multiple diseases	0.92 (0.81–1.06)	0.254		

## 4 Discussion

### 4.1 The prevalence of borderline elevated and elevated tc levels among newly hospitalized adult patients in jiangsu province was lower than the national average

Our study found that the overall prevalence of borderline-elevated and elevated TC among newly admitted adults in Jiangsu Province was 24.9%. Elevated TC is a well-established contributor to systemic morbidity and premature mortality (9) and a major risk factor for cardiovascular disease; it can promote aortic valve calcification and stenosis, which is associated with adverse coronary outcomes (10). Age-standardized coronary heart disease (CHD) mortality in the United States began to decline from 1968. Between 1980 and 2000, CHD mortality fell by over 40%; roughly 44% of this reduction was attributable to risk-factor modification, the single largest contribution (24%) arising from reductions in TC (11). A study conducted from 2007 to 2008, based on a nationally representative sample of individuals aged 20 years and older, found that the overall prevalence of borderline elevated TC and elevated cholesterol, was 31.5%. (12). Regional studies in China have reported higher estimates in some settings: Shandong Province (2011–2012, adults ≥40 years) 56.02% (13), Shenzhen (2011, adults >35 years) 46.65% (14), and Beijing (2008, adults 18–79 years) 31.2% (15). The prevalence of borderline-elevated and elevated TC among newly hospitalized adult patients in Jiangsu Province was lower than the national average, suggesting a potential association with the historically preserved Jiangnan dietary pattern in the region. The Jiangnan dietary pattern, as a representative of the Eastern healthy dietary pattern in the 2022 “Chinese Dietary Guidelines,” is characterized by mild seasoning, balanced nutrition, and refined cooking methods (16). The key dietary points are as follows: (1) a high intake of vegetables and fruits to achieve a low-sodium and high-potassium dietary pattern; (2) a focus on whole grains, replacing refined grains; (3) the use of rapeseed oil as the primary fat source, as it is rich in monounsaturated and polyunsaturated fatty acids; (4) a diet primarily based on white meat, with a significant consumption of seafood. Fish, especially marine fish, are rich in unsaturated fatty acids, which play an important role in lowering blood lipid levels (17). Rapeseed has been cultivated in the Jiangnan region for thousands of years, and residents of the southeastern coastal areas have traditionally used cold-pressed rapeseed oil. This oil has a high polyunsaturated-to-saturated fat ratio (rich in omega-6), and its protective effects on reducing overall mortality are similar to those of oils rich in monounsaturated fats (18). Study had shown that the Jiangnan dietary pattern can improve the plasma lipid profile (19). And some studies suggest that the Jiangnan diet may help prevent and control cardiovascular diseases (CVD) (20). These findings collectively suggest that the Jiangnan diet has a positive effect on chronic non-communicable diseases.

### 4.2 The prevalence of borderline and elevated TC increased with age and followed a U-shaped curve, first rising and then decreasing

Our study found that the prevalence of borderline elevated TC and elevated TC exhibited an inverted U-shaped curve, initially increasing with age and then decreasing. TC levels rose steadily during early life, reaching a peak before gradually declining (21, 22). The impact of age on lipid levels may be related to factors such as decreased food intake, impaired digestive absorption, and reduced hepatic synthesis function in older adults. Additionally, it may be attributable to survival selection, where elderly individuals may possess certain genetic traits that enable them to prolong their lives and combat the effects of physiological decline (23). A recently published study used the concentrations of cholesterol precursors (cholestenol, desmosterol, and lathosterol) as markers of cholesterol absorption and reported reduced cholesterol synthesis and decreased cholesterol absorption efficiency in an elderly cohort, thereby providing additional evidence to help explain the potential mechanisms underlying lower cholesterol levels observed in older adults (24). The literature related to the “cholesterol paradox” suggests that excessively low TC levels may increase the risk of all-cause mortality in older adults (25). A study published in 2017 followed over 3,000 elderly individuals in Sweden with an average follow-up duration of approximately 7.5 years. The results indicated that elderly individuals with “borderline-elevated” and “elevated” cholesterol levels had a significantly lower risk of mortality compared to those with normal cholesterol levels, with a reduction in risk of 29% and 32%, respectively (26). Therefore, low TC levels in individuals aged over 80 years do not necessarily reflect a healthy condition. Several guidelines (8, 27, 28) have established specific lipid management recommendations for the elderly population aged 75 years and older. European and Chinese lipid guidelines further suggest increasing the frequency of lipid screening after critical age thresholds; in Europe, this was recommended for men over 40 years and women over 50 years or post-menopause, while in China, it is suggested for those over 40 years. However, there was inconsistency in the definition of critical age and a lack of supporting evidence. Our research indicated that the prevalence of borderline elevated and elevated TC began to increase significantly after the age of 35. Therefore, we recommended initiating screening at the age of 35, with a particular focus on younger populations.

### 4.3 The risk of borderline and elevated tc was higher in women

Our study found a significantly higher prevalence of borderline-elevated and elevated TC in women than in men. This observation aligned with prior reports from rural China demonstrating higher TC prevalence among middle-aged women (29). The Framingham Study further showed that, after adjustment for age, mean TC increased from 220 to 237 mg/dL in postmenopausal compared with premenopausal women (30). Consistently, the SWAN study documented a significant rise in LDL-C around the final menstrual period, from 116 mg/dL (95% CI, 113–118) in the year before menopause to 128 mg/dL (95% CI, 125–131) in the year after, a change independent of chronological aging and ethnicity (31). A South Korean study of nearly 1,000 women likewise reported a marked increase in dyslipidaemia prevalence, with estimates rising from 28 to 56% across analyzed age strata (32). Estrogen deficiency during the menopausal transition was a major contributor to adverse cardiometabolic changes, including unfavorable shifts in lipid profiles and preferential central fat accumulation, which together increase cardiovascular risk (33). In recognition of this, the 2020 AHA scientific statement on the menopausal transition characterized this period as one of accelerated cardiovascular risk and advocates proactive lipid management (34). Our findings supported heightened emphasis on dyslipidaemia prevention and control in women.

### 4.4 The prevalence of borderline and elevated tc was lower in Nanjing

Our findings indicated that the prevalence of borderline-elevated and elevated TC in Nanjing was lower than in many other regions. Previous studies showed that awareness, treatment and control of dyslipidaemia were lowest among low-income populations (35). This disparity likely reflected advantages of provincial capitals such as Nanjing, where superior healthcare infrastructure and higher socioeconomic development facilitate more effective lipid management. A 2020 Nature analysis synthesized data from 1,127 population-based studies (102.6 million adults aged ≥18 years) to estimate TC trends in 200 countries from 1980 to 2018. The largest declines in TC occurred in high-income Western regions and Central and Eastern Europe; Northern Europe had the highest TC in 1980 and experienced the greatest reduction (~0.3 mmol/L per decade; posterior probability >0.9999). Most other regions showed minimal average change, whereas several low- and middle-income regions—particularly East and Southeast Asia—exhibited rises in TC exceeding 0.1 mmol/L per decade in both women and men (posterior probability >0.95) (36). Similar patterns have been reported in Lancet and JAMA, which support our observations (37, 38). These regional differences likely reflected variation in dietary policies, public-health strategies and access to lipid-lowering therapies (37).

### 4.5 The risk of borderline and elevated tc was lower in patients with nervous system diseases

We observed that patients with nervous system diseases had lower odds of borderline-elevated and elevated TC. However, the association between TC and stroke remains controversial. Historically, high TC has been regarded as a stroke risk factor, but recent studies report heterogeneous findings. The Kailuan Study in China found that very low TC ( ≤ 1.06 mmol/L) was associated with increased risks of total stroke, ischemic stroke and haemorrhagic stroke in both men and women (39). Yurong Zhang (40) reported a positive association between TC and stroke risk in men, but an inverse association with intracerebral hemorrhage in women. Kurth et al. (41) also described a positive relationship between TC and stroke risk. Several investigations suggested opposing associations by stroke subtype: elevated TC was more strongly linked to ischaemic stroke, whereas low TC was associated with higher risk of intracerebral hemorrhage (42). Our study suggested that the associated risk was relatively low, which may be attributed to the selection of subjects primarily consisting of patients with cerebral hemorrhage rather than those with ischemic stroke. Future studies should stratify stroke subtypes and clarify the underlying mechanisms to resolve these divergent observations.

### 4.6 The risk of borderline and elevated tc was lower in patients with diseases of the circulatory system

Our study found that patients with circulatory diseases had a lower risk of borderline-elevated and elevated TC. Ravnskov U (43) suggested that high TC may not promote atherosclerosis and might even be protective, possibly through favorable modulation of the immune system. A Lancet report in 2,000 indicated that low cholesterol in patients with oedematous chronic heart failure was associated with poorer perioperative and long-term survival (44). Similarly, Luo W et al. (45) observed significantly lower TC in patients with myocardial infarction due to coronary atherosclerosis than in healthy controls. These observations were consistent with our results and warrant further investigation to clarify the underlying causal mechanisms.

### 4.7 The risk of borderline and elevated tc remained unchanged in patients with endocrine, nutritional, and metabolic disorders

Common endocrine, nutritional and metabolic disorders encompass conditions such as diabetes mellitus, obesity and hyperuricaemia. Although the mechanisms linking glucose and lipid metabolism remain incompletely understood, epidemiological studies reported a close association between glycaemia and lipid profiles, including a positive correlation between TC and fasting plasma glucose (FPG) (46). At a mechanistic level, intracellular cholesterol accumulation may impair glucose metabolism by disrupting the translocation and activation of glucokinase (47). Cholesterol also preferentially accumulates in insulin-secretory granules of β-cells; excessive islet cholesterol causes β-cell dysfunction, impairs insulin secretion and increases β-cell apoptosis (48). Moreover, the development of hyperuricaemia has been positively associated with elevated TC (49). The results of this study indicate that participants with endocrine, nutritional, and metabolic disorders had a significantly higher prevalence of borderline-elevated and elevated TC levels, consistent with previous reports.

The strengths of our study are as follows. First, this research was based on a multicenter cluster sampling method, which minimizes sampling error and enhances representativeness and generalizability. Second, our study provides an evidence-based rationale and practical guidance for standardized dyslipidaemia management and intervention, informing related policy development. Notably, it was the first to propose lowering the recommended screening age to 35 years. Third, this study represented the latest evidence regarding the prevalence of borderline elevated TC and elevated TC in Jiangsu Province, filling a gap in the epidemiological trends of these conditions among adult inpatients over the past decade. However, this study had several limitations. First, the participating centers were predominantly tertiary hospitals, and excluding secondary hospitals that lacked clinical nutrition departments may have led to underrepresentation of some hospitalized patients. Second, the exclusion of patients with psychiatric disorders, cognitive impairment, critical illness, or insufficient capacity for appropriate behavior may have introduced selection bias and reduced the generalisability of the findings.

In summary, among newly hospitalized adults in Jiangsu Province, the prevalence of borderline-elevated and elevated TC followed an inverted U-shaped trajectory with age and was lower than the national average. Prevalence was also significantly associated with sex, region and the presence of neurological or circulatory comorbidities.

## Data Availability

The original contributions presented in the study are included in the article/Supplementary material, further inquiries can be directed to the corresponding authors.
