# Supplementary material for: Prevalence of borderline elevated and elevated cholesterol among new adult patients from 23 hospitals in 12 cities of Jiangsu Province: a multicenter cross-sectional study
Source: Front Nutr. 2026 Apr 1;13:1800853. doi: 10.3389/fnut.2026.1800853 (PMC13078996; doi:10.3389/fnut.2026.1800853)
Supplement: Supplementary file 1 [file Table_1.docx]

Table S1 Age-Standardized Prevalence Rates of Borderline Elevated Total Cholesterol Across Different Regions

| **Region** | **Crude Prevalence (%)** | **Age-Standardized Prevalence (%)** | **95% CI (%)** |
| --- | --- | --- | --- |
| Nanjing Area | 19.9 | 19.1 | 17.0 – 21.2 |
| Southern Jiangsu | 26.6 | 25.8 | 23.2 – 28.4 |
| Central Jiangsu | 26.7 | 27.5 | 24.7 – 30.3 |
| Northern Jiangsu | 26.6 | 28.1 | 25.1 – 31.1 |
| Overall | 25 | 24.9 | 23.5 – 26.3 |
